# Supplementary material for: HoBi-like Pestivirus Is Highly Prevalent in Cattle Herds in the Amazon Region (Northern Brazil)
Source: Viruses. 2023 Feb 6;15(2):453. doi: 10.3390/v15020453 (PMC9965828; doi:10.3390/v15020453)
Supplement: Supplementary file 1 [file viruses-15-00453-s001.zip › Suplementary Table S3.pdf]

Table S3: Individual results for VNT titers for BVDV-1, 2 and HoBiPeV.

			VNT titers**		
**Sample ID**	**State***	**City**	**BVDV-1a (strain C24V)**	**BVDV-2b (strain SV253)**	**HoBiPeV (strain Italy 83/10)**
1	RR	Amajari	No Ab	No Ab	No Ab
2	RR	Amajari	512	No Ab	2048
3	RR	Amajari	No Ab	No Ab	No Ab
4	RR	Amajari	128	16	128
5	RR	Amajari	128	No Ab	No Ab
6	RR	Alto Alegre	256	32	No Ab
7	RR	Alto Alegre	32	No Ab	No Ab
8	RR	Alto Alegre	No Ab	No Ab	No Ab
9	RR	Alto Alegre	No Ab	No Ab	No Ab
10	RR	Alto Alegre	No Ab	No Ab	No Ab
11	RR	Alto Alegre	No Ab	128	No Ab
12	RR	Alto Alegre	32	128	No Ab
13	RR	Alto Alegre	No Ab	64	No Ab
14	RR	Alto Alegre	No Ab	64	No Ab
15	RR	Boa Vista	16	64	128
16	RR	Boa Vista	No Ab	32	64
17	RR	Bonfim	No Ab	No Ab	No Ab
18	RR	Bonfim	No Ab	No Ab	No Ab
19	RR	Bonfim	No Ab	No Ab	No Ab
20	RR	Bonfim	256	512	128
21	RR	Cantá	No Ab	32	No Ab
22	RR	Cantá	No Ab	128	No Ab
23	RR	Cantá	32	1024	128
24	RR	Cantá	No Ab	32	No Ab
25	RR	Cantá	No Ab	32	No Ab
26	RR	Cantá	32	64	No Ab
27	RR	Cantá	32	No Ab	No Ab
28	RR	Caroebe	No Ab	No Ab	128
29	RR	Caroebe	No Ab	64	No Ab
30	RR	Caroebe	No Ab	No Ab	512

31	RR	Iracema	No Ab	No Ab	No Ab
32	RR	Caroebe	32	128	512
33	RR	Caroebe	No Ab	No Ab	No Ab
34	RR	Caroebe	No Ab	No Ab	No Ab
35	RR	Caroebe	No Ab	32	No Ab
36	RR	Iracema	512	64	512
37	RR	Iracema	256	128	1024
38	RR	Mucajaí	No Ab	8	No Ab
39	RR	Mucajaí	No Ab	32	512
40	RR	Normandia	256	512	64
41	RR	Normandia	No Ab	32	256
42	RR	Pacaraima	64	128	No Ab
43	RR	São João da Baliza	1024	128	No Ab
44	RR	São Luiz	128	512	No Ab
45	RR	São Luiz	No Ab	No Ab	No Ab
46	RR	São Luiz	No Ab	No Ab	No Ab
47	RR	Uiramutã	No Ab	No Ab	No Ab
48	PA	Afuá	No Ab	32	No Ab
49	PA	Breves	No Ab	16	No Ab
50	PA	Chaves	No Ab	No Ab	256
51	PA	Chaves	64	64	No Ab
52	PA	Chaves	64	No Ab	128
53	PA	Chaves	No Ab	No Ab	No Ab
54	PA	Chaves	No Ab	128	No Ab
55	PA	Chaves	256	1024	256
56	PA	Chaves	64	128	128
57	PA	Chaves	64	64	128
58	PA	Chaves	No Ab	128	No Ab
59	PA	Chaves	No Ab	No Ab	32
60	PA	Chaves	No Ab	16	No Ab
61	PA	Chaves	No Ab	No Ab	64
62	PA	Chaves	No Ab	512	No Ab
63	PA	Chaves	No Ab	16	128
64	PA	Chaves	64	No Ab	128
65	PA	Chaves	64	No Ab	No Ab
66	PA	Chaves	No Ab	No Ab	No Ab

67	PA	Gurupá	16	No Ab	8
68	PA	Gurupá	No Ab	No Ab	No Ab
69	PA	Gurupá	No Ab	512	No Ab
70	PA	Melgaço	No Ab	512	512
71	RR	Amajari	8	No Ab	No Ab
72	RR	Boa Vista	16	No Ab	No Ab
73	RR	Bonfim	32	128	No Ab
74	RR	Bonfim	32	16	No Ab
75	RR	Cantá	128	128	No Ab
76	RR	Cantá	No Ab	128	No Ab
77	RR	Caracaraí	64	128	8
78	AM	Envira	32	No Ab	No Ab
79	RR	Caracaraí	256	64	No Ab
80	RR	Caracaraí	No Ab	32	32
81	RR	Caroebe	64	128	No Ab
82	RR	Caroebe	128	32	No Ab
83	RR	Caroebe	No Ab	No Ab	16
84	RR	Iracema	No Ab	No Ab	No Ab
85	RR	Iracema	No Ab	No Ab	No Ab
86	RR	Mucajaí	No Ab	No Ab	No Ab
87	RR	Mucajaí	No Ab	No Ab	No Ab
88	RR	Mucajaí	16	16	128
89	RR	Mucajaí	128	No Ab	No Ab
90	RR	Mucajaí	32	No Ab	No Ab
91	RR	Mucajaí	No Ab	No Ab	No Ab
92	RR	Normandia	No Ab	No Ab	No Ab
93	RR	Rorainópolis	128	No Ab	8
94	RR	Rorainópolis	64	256	512
95	RR	Rorainópolis	256	256	512
96	RR	Rorainópolis	64	No Ab	No Ab
97	RR	Rorainópolis	1024	No Ab	No Ab
98	RR	Rorainópolis	16	No Ab	No Ab
99	RR	São João da Baliza	32	No Ab	No Ab
100	RR	São João da Baliza	128	No Ab	No Ab
101	RR	São Luiz	64	No Ab	No Ab
102	RR	São Luiz	No Ab	No Ab	No Ab

103	AP	Cutias	64	No Ab	No Ab
104	AP	Cutias	32	No Ab	No Ab
105	AP	Cutias	16	No Ab	No Ab
106	AP	Cutias	512	No Ab	512
107	AP	Ferreira Gomes	256	128	No Ab
108	AP	Itaubal	No Ab	64	No Ab
109	AP	Macapá	512	32	No Ab
110	AP	Macapá	No Ab	No Ab	No Ab
111	AP	Macapá	256	64	No Ab
112	AP	Macapá	16	No Ab	No Ab
113	AP	Macapá	32	64	No Ab
114	AP	Mazagão	No Ab	No Ab	No Ab
115	AP	Mazagão	No Ab	256	No Ab
116	AP	Porto Grande	256	256	1024
117	AP	Santana	No Ab	No Ab	No Ab
118	AP	Tartarugalzinho	No Ab	8	No Ab
119	AP	Tartarugalzinho	No Ab	No Ab	No Ab
120	AP	Tartarugalzinho	No Ab	No Ab	16
121	AP	Tartarugalzinho	No Ab	No Ab	8
122	AP	Vitória do Jari	No Ab	No Ab	8
123	AP	Vitória do Jari	No Ab	16	No Ab
124	PA	Faro	No Ab	No Ab	8
125	PA	Terra Santa	No Ab	32	64
126	PA	Terra Santa	512	256	512
127	PA	Terra Santa	No Ab	64	8
128	PA	Terra Santa	No Ab	No Ab	No Ab
129	PA	Terra Santa	No Ab	No Ab	No Ab
130	AP	Amapá	No Ab	32	No Ab
131	AP	Amapá	No Ab	8	8
132	AP	Amapá	No Ab	No Ab	8
133	AP	Amapá	512	2048	1024
134	AP	Pedra Branca do Amapari	No Ab	No Ab	No Ab
135	AP	Cutias	No Ab	No Ab	No Ab
136	AP	Cutias	128	128	512
137	AP	Itaubal	No Ab	No Ab	8
138	AP	Laranjal do Jari	No Ab	No Ab	No Ab

139	AP	Macapá	No Ab	No Ab	16
140	AP	Macapá	No Ab	No Ab	No Ab
141	AP	Macapá	No Ab	No Ab	No Ab
142	AP	Macapá	No Ab	No Ab	8
143	AP	Macapá	No Ab	No Ab	No Ab
144	AP	Macapá	No Ab	No Ab	No Ab
145	AP	Macapá	No Ab	No Ab	No Ab
146	AP	Macapá	No Ab	No Ab	No Ab
147	AP	Macapá	No Ab	32	128
148	AP	Mazagão	No Ab	No Ab	No Ab
149	AP	Mazagão	No Ab	No Ab	No Ab
150	AP	Porto Grande	No Ab	No Ab	No Ab
151	AP	Porto Grande	No Ab	No Ab	No Ab
152	AP	Santana	256	128	256
153	AP	Tartarugalzinho	No Ab	No Ab	No Ab
154	AP	Tartarugalzinho	1024	256	256
155	AP	Tartarugalzinho	512	1024	2048
156	AP	Tartarugalzinho	No Ab	No Ab	No Ab
157	AP	Tartarugalzinho	1024	512	2048
158	AP	Tartarugalzinho	No Ab	No Ab	No Ab
159	AP	Tartarugalzinho	No Ab	No Ab	8
160	AP	Vitória do Jari	No Ab	No Ab	8
161	AP	Amapá	No Ab	No Ab	No Ab
162	AP	Amapá	No Ab	No Ab	No Ab
163	AP	Laranjal do Jari	No Ab	No Ab	No Ab
164	AP	Macapá	No Ab	No Ab	No Ab
165	AP	Vitória do Jari	No Ab	No Ab	No Ab
166	AM	Santa Isabel do Rio Negro	No Ab	No Ab	No Ab
167	AM	Carauari	No Ab	No Ab	8
168	AM	Carauari	No Ab	No Ab	8
169	AM	Carauari	No Ab	No Ab	No Ab
170	AM	Carauari	No Ab	No Ab	16
171	AM	Carauari	No Ab	No Ab	No Ab
172	AM	Envira	No Ab	No Ab	No Ab
173	AM	Envira	No Ab	No Ab	8
174	AM	Envira	No Ab	No Ab	8

175	AM	Envira	512	256	512
176	AM	Envira	512	64	1024
177	AM	Envira	No Ab	No Ab	No Ab
178	AM	Envira	No Ab	No Ab	No Ab
179	AM	Envira	No Ab	No Ab	No Ab
180	AM	Envira	No Ab	No Ab	No Ab
181	AM	Envira	No Ab	128	128
182	AM	Envira	No Ab	No Ab	8
183	AM	Envira	No Ab	No Ab	No Ab
184	AM	Envira	No Ab	No Ab	No Ab
185	AM	Envira	No Ab	No Ab	No Ab
186	AM	Envira	No Ab	No Ab	No Ab
187	AM	Envira	No Ab	No Ab	No Ab
188	AM	Humaitá	256	No Ab	No Ab
189	AM	Humaitá	No Ab	No Ab	No Ab
190	AM	Humaitá	No Ab	No Ab	No Ab
191	AM	Humaitá	No Ab	No Ab	No Ab
192	AM	Ipixuna	No Ab	No Ab	No Ab
193	AM	Ipixuna	No Ab	No Ab	No Ab
194	AM	Ipixuna	No Ab	No Ab	No Ab
195	AM	Ipixuna	No Ab	No Ab	No Ab
196	AM	Ipixuna	No Ab	No Ab	No Ab
197	AM	Ipixuna	No Ab	No Ab	No Ab
198	AM	Ipixuna	No Ab	No Ab	No Ab
199	AM	Ipixuna	No Ab	No Ab	No Ab
200	AM	Pauini	No Ab	No Ab	No Ab
201	AM	Pauini	No Ab	No Ab	No Ab
202	AM	Pauini	No Ab	No Ab	No Ab
203	AM	Pauini	No Ab	No Ab	No Ab
204	AM	Pauini	No Ab	No Ab	No Ab
205	AM	Coari	No Ab	No Ab	No Ab
206	AM	Coari	No Ab	No Ab	No Ab
207	AM	Iranduba	No Ab	No Ab	No Ab
208	AM	Iranduba	No Ab	No Ab	No Ab
209	AM	Juruá	No Ab	No Ab	No Ab
210	AM	Juruá	No Ab	No Ab	No Ab

211	AM	Manicoré	No Ab	No Ab	No Ab
212	AM	Manicoré	No Ab	No Ab	No Ab
213	AM	Manicoré	No Ab	No Ab	No Ab
214	AM	Manicoré	No Ab	No Ab	No Ab
215	AM	Manicoré	128	128	128
216	AM	Manicoré	No Ab	No Ab	No Ab
217	AM	Manicoré	No Ab	No Ab	No Ab
218	AM	Manicoré	No Ab	No Ab	No Ab
219	AM	Manicoré	512	256	2048
220	AM	Manicoré	No Ab	No Ab	No Ab
221	AM	Manicoré	No Ab	No Ab	No Ab
222	AM	Manicoré	No Ab	No Ab	No Ab
223	AM	Manicoré	No Ab	No Ab	No Ab
224	AM	Manicoré	256	512	No Ab
225	AM	Manicoré	No Ab	No Ab	No Ab
226	AM	Manicoré	No Ab	No Ab	No Ab
227	AM	Manicoré	32	512	2048
228	AM	Manicoré	128	No Ab	No Ab
229	AM	Manicoré	No Ab	No Ab	No Ab
230	AM	Manicoré	No Ab	No Ab	8
231	AM	Manicoré	256	256	512
232	AM	Manicoré	512	64	512
233	AM	Manicoré	No Ab	No Ab	No Ab
234	AM	Manicoré	512	16	32
235	AM	Manicoré	No Ab	No Ab	No Ab
236	AM	Manicoré	1024	256	2048
237	AM	Presidente Figueiredo	512	256	2048
238	AM	Presidente Figueiredo	No Ab	No Ab	No Ab
239	AM	Rio Preto da Eva	No Ab	No Ab	No Ab
240	AM	Tapauá	No Ab	No Ab	No Ab
241	AM	Tapauá	No Ab	No Ab	No Ab
242	AM	Apuí	No Ab	No Ab	No Ab
243	AM	Apuí	1024	128	No Ab
244	AM	Apuí	No Ab	No Ab	No Ab
245	AM	Apuí	No Ab	No Ab	No Ab
246	AM	Apuí	No Ab	No Ab	No Ab

247	AM	Apuí	No Ab	No Ab	No Ab
248	AM	Apuí	No Ab	No Ab	No Ab
249	AM	Apuí	16	64	128
250	AM	Apuí	No Ab	No Ab	No Ab
251	AM	Apuí	8	No Ab	No Ab
252	AM	Apuí	No Ab	No Ab	No Ab
253	AM	Apuí	No Ab	No Ab	8
254	AM	Apuí	No Ab	No Ab	8
255	AM	Apuí	No Ab	No Ab	No Ab
256	AM	Apuí	No Ab	No Ab	No Ab
257	AM	Apuí	256	2048	1024
258	AM	Apuí	No Ab	No Ab	No Ab
259	AM	Barreirinha	No Ab	No Ab	No Ab
260	AM	Barreirinha	No Ab	No Ab	No Ab
261	AM	Barreirinha	128	256	2048
262	AM	Barreirinha	No Ab	No Ab	No Ab
263	AM	Boa Vista do Ramos	No Ab	No Ab	No Ab
264	AM	Boa Vista do Ramos	No Ab	No Ab	No Ab
265	AM	Boa Vista do Ramos	No Ab	No Ab	No Ab
266	AM	Boa Vista do Ramos	No Ab	No Ab	No Ab
267	AM	Caapiranga	No Ab	No Ab	No Ab
268	AM	Eirunepé	No Ab	No Ab	No Ab
269	AM	Eirunepé	No Ab	No Ab	No Ab
270	AM	Eirunepé	No Ab	No Ab	No Ab
271	AM	Eirunepé	No Ab	No Ab	No Ab
272	AM	Eirunepé	No Ab	No Ab	No Ab
273	AM	Eirunepé	No Ab	No Ab	No Ab
274	AM	Fonte Boa	No Ab	No Ab	8
275	AM	Fonte Boa	No Ab	No Ab	No Ab
276	AM	Fonte Boa	No Ab	No Ab	No Ab
277	AM		64	No Ab	No Ab
278	AM	Itamarati	No Ab	No Ab	No Ab
279	AM	Itamarati	No Ab	No Ab	No Ab
280	AM	Borba	No Ab	No Ab	No Ab
281	AM	Borba	No Ab	No Ab	No Ab
282	AM	Borba	256	128	2048

283	AM	Canutama	No Ab	No Ab	No Ab
284	AM	Canutama	No Ab	No Ab	No Ab
285	AM	Canutama	No Ab	No Ab	No Ab
286	AM	Codajás	16	256	256
287	AM	Codajás	No Ab	No Ab	No Ab
288	AM	Codajás	No Ab	No Ab	No Ab
289	AM	Codajás	No Ab	No Ab	No Ab
290	AM	Itapiranga	No Ab	No Ab	No Ab
291	AM	Itapiranga	No Ab	No Ab	No Ab
292	AM	Maués	No Ab	No Ab	No Ab
293	AM	Maués	No Ab	No Ab	No Ab
294	AM	Maués	No Ab	No Ab	No Ab
295	AM	Nhamundá	No Ab	No Ab	No Ab
296	AM	Nhamundá	No Ab	No Ab	No Ab
297	AM	Nova Olinda do Norte	No Ab	No Ab	No Ab
298	AM	Nova Olinda do Norte	No Ab	No Ab	No Ab
299	AM	Nova Olinda do Norte	No Ab	No Ab	No Ab
300	AM	Parintins	No Ab	No Ab	No Ab
301	AM	Parintins	No Ab	No Ab	No Ab
302	AM	Parintins	No Ab	No Ab	No Ab
303	AM	Parintins	No Ab	No Ab	No Ab
304	AM	Parintins	64	32	512
305	AM	Parintins	No Ab	No Ab	No Ab
306	AM	Parintins	No Ab	No Ab	8
307	AM	Parintins	16	No Ab	No Ab
308	AM	Parintins	No Ab	No Ab	No Ab
309	AM	São Sebastião do Uatumã	No Ab	No Ab	No Ab
310	AM	Silves	No Ab	No Ab	No Ab
311	AM	Silves	No Ab	No Ab	No Ab
312	AM	Silves	No Ab	No Ab	No Ab
313	AM	Urucará	No Ab	No Ab	8
314	AM	Autazes	No Ab	No Ab	No Ab
315	AM	Autazes	No Ab	No Ab	No Ab
316	AM	Autazes	No Ab	No Ab	8
317	AM	Autazes	8	64	No Ab
318	AM	Autazes	No Ab	No Ab	No Ab

319	AM	Autazes	No Ab	8	No Ab
320	AM	Autazes	No Ab	No Ab	No Ab
321	AM	Autazes	No Ab	No Ab	No Ab
322	AM	Autazes	No Ab	No Ab	No Ab
323	AM	Autazes	No Ab	32	8
324	AM	Autazes	No Ab	No Ab	No Ab
325	AM	Autazes	No Ab	No Ab	No Ab
326	AM	Autazes	No Ab	64	8
327	AM	Autazes	No Ab	No Ab	No Ab
328	AM	Autazes	No Ab	No Ab	8
329	AM	Autazes	No Ab	No Ab	8
330	AM	Autazes	32	No Ab	No Ab
331	AM	Autazes	No Ab	No Ab	No Ab
332	AM	Autazes	No Ab	1024	1024
333	AM	Autazes	No Ab	No Ab	No Ab
334	AM	Autazes	8	No Ab	No Ab
335	AM	Autazes	32	No Ab	No Ab
336	AM	Autazes	No Ab	No Ab	32
337	AM	Autazes	No Ab	256	128
338	AM	Autazes	No Ab	No Ab	No Ab
339	AM	Autazes	No Ab	No Ab	No Ab
340	AM	Itacoatiara	No Ab	No Ab	8
341	AM	Itacoatiara	No Ab	256	2048
342	AM	Itacoatiara	No Ab	No Ab	No Ab
343	AM	Itacoatiara	No Ab	No Ab	No Ab
344	AM	Itacoatiara	No Ab	No Ab	8
345	AM	Itacoatiara	No Ab	No Ab	No Ab
346	AM	Itacoatiara	128	No Ab	No Ab
347	AM	Itacoatiara	16	64	No Ab
348	AM	Itacoatiara	No Ab	No Ab	No Ab
349	AM	Tefé	No Ab	16	No Ab
350	AM	Amaturá	No Ab	No Ab	No Ab
351	AM	Anamã	No Ab	No Ab	No Ab
352	AM	Careiro	No Ab	No Ab	No Ab
353	AM	Careiro	No Ab	No Ab	No Ab
354	AM	Careiro	No Ab	No Ab	No Ab

355	AM	Careiro	No Ab	No Ab	No Ab
356	AM	Careiro	8	No Ab	No Ab
357	AM	Careiro	64	No Ab	No Ab
358	AM	Careiro	No Ab	No Ab	No Ab
359	AM	Careiro	No Ab	No Ab	No Ab
360	AM	Careiro da Várzea	128	256	512
361	AM	Itacoatiara	No Ab	No Ab	No Ab
362	AM	Careiro da Várzea	No Ab	No Ab	No Ab
363	AM	Careiro	256	64	512
364	AM	Careiro	32	No Ab	No Ab
365	AM	Careiro da Várzea	No Ab	No Ab	No Ab
366	AM	Manaus	1024	256	1024
367	AM	Careiro da Várzea	No Ab	No Ab	No Ab
368	AM	Autazes	No Ab	No Ab	No Ab
369	AM	Japurá	No Ab	No Ab	No Ab
370	AM	Japurá	No Ab	No Ab	No Ab
371	AM	Lábrea	No Ab	No Ab	No Ab
372	AM	Lábrea	No Ab	No Ab	8
373	AM	Lábrea	No Ab	No Ab	No Ab
374	AM	Lábrea	No Ab	No Ab	No Ab
375	AM	Lábrea	No Ab	No Ab	No Ab
376	AM	Lábrea	No Ab	No Ab	No Ab
377	AM	Lábrea	No Ab	No Ab	No Ab
378	AM	Manacapuru	No Ab	No Ab	No Ab
379	AM	Manacapuru	No Ab	No Ab	No Ab
380	AM	Manaquiri	No Ab	No Ab	No Ab
381	AM	Manaquiri	No Ab	No Ab	No Ab
382	AM	Santo Antônio do Içá	No Ab	No Ab	No Ab
383	AM	Tabatinga	No Ab	No Ab	No Ab
384	AM	Tonantins	No Ab	No Ab	No Ab
385	AM	Tonantins	No Ab	No Ab	32
386	AM	Urucurituba	No Ab	No Ab	No Ab
387	AM	Urucurituba	No Ab	No Ab	No Ab
388	AM	Urucurituba	No Ab	No Ab	No Ab
389	AM	Urucurituba	No Ab	No Ab	No Ab
390	AM	Urucurituba	No Ab	No Ab	No Ab

* Brazil state: RR- Roraima; PA- Pará; AP- Amapá; AM- Amazonas.
** Titers <8 are considered negative (No Ab).
