# Supplementary material for: HoBi-like Pestivirus Is Highly Prevalent in Cattle Herds in the Amazon Region (Northern Brazil)
Source: Viruses. 2023 Feb 6;15(2):453. doi: 10.3390/v15020453 (PMC9965828; doi:10.3390/v15020453)
Supplement: Supplementary file 1 [file viruses-15-00453-s001.zip › Supplementary Materials.pdf]

Supplementary Materials:

Table S1: Number of cattle (in million heads) in different regions of Brazil

Region	Federative State	(n) million heads
NORTHERN	Rondônia	15.1
	Acre	4.1
	Amazonas	1.5
	Roraima	0.9
	Pará	23.9
	Amapá	0.05
	Tocantins	10.1
**Total**		**55.6**
NORTHEAST	Maranhão	8.5
	Piauí	1.4
	Ceará	2.6
	Rio Grande do Norte	1.1
	Paraíba	1.3
	Pernambuco	2.2
	Alagoas	1.3
	Sergipe	1.1
	Bahia	11.7
**Total**		**31.2**
CENTRAL-WEST	Mato Grosso do Sul	18.6
	Mato Grosso	32.4
	Goiás	24.3
	Distrito Federal	0.08
**Total**		**75.4**
SOUTHEAST	Minas Gerais	22.9
	Espírito Santo	2.2
	Rio de Janeiro	2.7
	São Paulo	10.7
**Total**		**38.5**
SOUTH	Paraná	8.1
	Santa Catarina	4.5
	Rio Grande do Sul	11.1
**Total**		**23.7**

References: [35,36].

Table S2: Percentage of positive samples by titer for BVDV-1, 2 and HoBiPeV

Titer*	BVDV-1 (%)	BVDV-2 (%)	HoBiPeV (%)
8 to <16	5	3	32
16 to <64	28	25	9
64 to <256	30	40	20
256 to < 1,024	30	25	22
≥1,024	7	5	17
**Total**	**100**	**100**	**100**

* Titers 8 to <16 are considered low. Titers 16-128 are considered moderate. Titers ≥ 256 are considered high.

Table S3: Individual results for VNT titers for BVDV-1, 2 and HoBiPeV. (View excel.xls file)

Table S4: The major countries live cattle exporters worldwide.

Country	% Of world
Mexico	24.9%
European Union	22.9%
Australia	18.1%
Canada	13.4%
Brazil	8.5%
Uruguay	6.2%

References: [36].
